# Relationship between Immunological Abnormalities in Rat Models of Diabetes Mellitus and the Amplification Circuits for Diabetes

**DOI:** 10.1155/2017/4275851

**Published:** 2017-02-19

**Authors:** Yuji Takeda, Tomoko Shimomura, Hironobu Asao, Ichiro Wakabayashi

**Affiliations:** ^1^Department of Environmental and Preventive Medicine, Hyogo College of Medicine, Nishinomiya, Japan; ^2^Department of Immunology, Faculty of Medicine, Yamagata University, Yamagata, Japan

## Abstract

A better understanding of pathogenic mechanisms is required in order to treat diseases. However, the mechanisms of diabetes mellitus and diabetic complications are extremely complex. Immune reactions are involved in the pathogenesis of diabetes and its complications, while diabetes influences immune reactions. Furthermore, both diabetes and immune reactions are influenced by genetic and environmental factors. To address these issues, animal models are useful tools. So far, various animal models of diabetes have been developed in rats, which have advantages over mice models in terms of the larger volume of tissue samples and the variety of type 2 diabetes models. In this review, we introduce rat models of diabetes and summarize the immune reactions in diabetic rat models. Finally, we speculate on the relationship between immune reactions and diabetic episodes. For example, diabetes-prone Biobreeding rats, type 1 diabetes model rats, exhibit increased autoreactive cellular and inflammatory immune reactions, while Goto-Kakizaki rats, type 2 diabetes model rats, exhibit increased Th2 reactions and attenuation of phagocytic activity. Investigation of immunological abnormalities in various diabetic rat models is useful for elucidating complicated mechanisms in the pathophysiology of diabetes. Studying immunological alterations, such as predominance of Th1/17 or Th2 cells, humoral immunity, and innate immune reactions, may improve understanding the structure of amplification circuits for diabetes in future studies.

## 1. Introduction

The prevalence of diabetes mellitus (DM) is increasing in developed countries, including Japan. Obesity, which is caused by abnormalities in lifestyles, such as diet, nutrition, and physical activity, is the most important risk factor for type 2 diabetes mellitus. Obesity, a determinant in the pathogenesis of diabetes and diabetic complications, is also associated with various immune reactions in patients with diabetes. An accumulation of information on immune reactions associated with diabetic episodes is required in order to achieve a better understanding of its pathogenic mechanisms. Animal models of DM are useful for investigating the relationship between immune reactions and diabetic episodes.

In recent years, various type 2 DM models have been developed in rats. Rat models have the advantage over mice models that a higher volume of blood and tissue samples can be obtained from rats. For example, peripheral blood from rats can be collected in more than 10-fold volume as compared to blood from mice. For clinical examination, blood specimens from humans usually consist of peripheral blood, and therefore, the diabetic rat is a valuable experimental model for application to laboratory test. In this review, we summarize the diverse diabetic rat models and discuss the relationship between diabetic episodes and immune reactions.

## 2. Drug-Induced DM

Streptozotocin (STZ) and alloxan are often used for the induction of type 1 DM in animal models. These reagents cause the production of reactive oxygen species in the *β* cells of the pancreas, resulting in *β* cell death [1, 2]. Although these models of type 1 diabetes are useful for investigating the effect of high glucose conditions on immune responses without obesity, they are not suitable for investigation of autoimmune reactivity to *β* cells, which is known to be a major mechanism in the causation of type 1 diabetes in humans. Furthermore, Muller et al. clearly demonstrated that STZ both directly and indirectly induces suppressive effect on lymphocytes in mice [3]. The evaluation of immune responses in the drug-induced DM models must be carefully considered.

Alloxan also has a suppressive effect on lymphocytes [4]. However, Gaulton et al. reported that the effect of alloxan on lymphocytes is transient and concluded that alloxan-induced diabetes, but not STZ-induced diabetes, provides a useful model for evaluating immunological changes associated with hyperglycemia and diabetes [4]. Therefore, we focus on the alloxan-induced diabetes model in relation to immunity in this review.

The reason why only alloxan shows transient effect on lymphocyte remains unclear. The most likely reason is that the source of generated reactive oxygen species is different in the pharmacological action of alloxan and STZ. Alloxan is more unstable than STZ and is rapidly taken up into insulin-producing cells (*β* cells) and liver. The highly reactive hydroxyl radicals are formed by the Fenton reaction. However, the liver and other tissues are more resistant to reactive oxygen species in comparison to pancreatic *β* cells [1]. On the other hand, STZ enters into the cells* via* glucose transporter and then causes alkylation of DNA. DNA damage induces activation of poly-ADP-ribosylation, which leads to depletion of cellular NAD^+^ and ATP. Enhanced ATP dephosphorylation supplies a substrate for xanthine oxidase, resulting in the formation of superoxide radicals [1]. It is well known that T lymphocytes (T cells) also express the glucose transporter during proliferation [5] and that the cells are always accompanied with poly-ADP-ribosylation during proliferation [6, 7]. Therefore, inappropriate poly-ADP-ribosylation induced by STZ may be involved in irreversible impairment of T cell function.

In alloxan-induced diabetic rats, both cellular immunity and humoral immunity are impaired by hyperglycemia [4, 8]. Phagocytosis by alveolar macrophages from alloxan-DM rats is also impaired [9]. These findings are summarized in Table 1.

Impairment of both cellular and humoral immunity in DM was induced by dysfunction of lymphocyte proliferation, which is suppressed under high glucose conditions [8]. Impairment of phagocytosis is also induced by deficient coupling of the leukotriene receptor with the Fc*γ* receptor signaling cascade [9]. Stimulation with insulin to augment cell membrane kinetics plays a key role in this coupling [9]. These previous reports make it possible to propose that acquired immunity is regulated by glucose concentration and innate immunity is maintained by the secretion of insulin.

Recently, accumulating evidences have demonstrated that glucose is a key metabolic substrate for T cells as below [5]: naïve T cells mature and exit from the thymus primarily relying on oxidize glucose-derived pyruvate in their mitochondria via oxidative phosphorylation (OXPHOS) for their metabolic needs to generate cellular ATP. In the secondary lymphoid organs, memory T cells are a quiescent population of the cells that primarily use OXPHOS, but both OXPHOS and glycolysis increase rapidly after antigen rechallenge and facilitate their recall responses. On the other hand, chronic hyperglycemic conditions result in reduction of cellular ATP levels by O-GlcNAcylation of protein subunits of mitochondrial respiratory complexes I, III, and IV, intimately associated with normative cellular bioenergetics and ATP production [10]. Therefore, hyperglycemic conditions induce the dysfunction of mitochondrial respiration. These reports allow us to speculate that hyperglycemic condition with mitochondrial dysfunction may inhibit the primary T cell expansion and the memory T cell maintenance in the diabetic model rats.

STZ doses of 65~70 mg/kg are often used for inducing type 1 DM in rats. In some studies, lower doses of STZ, 30~35 mg/kg, were employed to create a type 2 DM model [11, 12]. Obese and hyperglycemic rats, such as high-fat diet-fed Sprague-Dawley (SD) rats and Zucker fatty rats, were treated with low doses of STZ in order to reduce insulin secretion. The exhaustion of *β* cells was artificially induced in these type 2 diabetes models. To our knowledge, there have been no reports on immunological observations using these low dose-STZ-induced diabetes models.

## 3. Type 1 DM Models

Type 1 DM, with a prevalence that is <5% of the prevalence of total DM cases, is considered an autoimmune disease. However, type 1 DM is known to occur through various pathological processes. For example, fulminant type 1 DM, which represents 20% of acute-onset type 1 DM, is not related to autoreactive immune responses [13]. Other pathological processes, such as slowly progressive insulin-dependent (type 1) diabetes mellitus (SPIDDM), or latent autoimmune diabetes in adults (LADA), which result from gradual distraction of islets of Langerhans in pancreas through an autoimmune response, are difficult to distinguish from type 2 DM [14, 15]. Thus, the pathological processes of type 1 DM are variable in humans.

Diabetes-prone Biobreeding (DP-BB) rats are a known type 1 DM-rat model. Th1 and Th17 responses are dominant, and moreover, the T cell receptor (TCR) repertoire is decreased in DP-BB rats due to T-lymphocytopenia [16]. These abnormalities trigger the onset of DM, as summarized in Table 1.

RT 1^u^ haplotype and a mutation in the* Gimap5* gene are known to be the genetic factors involved in the onset of diabetes in DP-BB rats [17, 18]. Gimap5 plays a key role in antiapoptosis and calcium signaling in T cells. Dysfunction of Gimap5 induces lymphopenia and inhibits the accumulation of a normal T cell pool, including regulatory subsets [19, 20]. Lymphopenia is considered a cause of an autoimmune reaction in DP-BB rats. On the other hand, diabetes resistant-BB (DR-BB) rats have a normal* Gimap5* gene. When T cells are modulated by irradiation, or when Th1 immune reactions are activated by virus infection, DR-BB rats also develop type 1 DM [21–23].

Recently, the inhibition of costimulatory signaling for T cell activation or transplantation of regulatory T cells has been shown to prevent the onset of DM using DP-BB rats [24–26]. In the future, repertoire or autoreactive antigen analysis of autoreactive T cells and regulatory T cells in DP-BB or DR-BB rats will be useful for the investigation of various pathological mechanisms in type 1 DM, such as fulminant type 1 DM, acute-onset type 1 DM, SPIDDM, and LADA type 1 DM.

## 4. Type 2 DM Model

### 4.1. Type 2 DM Model with Obesity

Obesity is a major risk factor for type 2 DM. The establishment and breeding of obese rats have been performed for a long time. Currently, the two main strains of obese model rats available are leptin receptor-deficient rats (Zucker fatty, ZF) and rats with a spontaneous lack of cholecystokinin 1 receptor (Otsuka Long-Evans Tokushima Fatty, OLETF) [27, 28].

Immunological investigations of type 2 diabetic rats have been performed extensively using Zucker rat strains. ZF rats exhibit susceptibility to infection, although phagocytosis by neutrophils and macrophages is not impaired [29, 30]. Furthermore, T-lymphocytopenia (CD4^+^ and CD8^+^ T cells) is observed in ZF rats [31, 32]. On the other hand, the production of immunoglobulins (IgA, IgG, and IgM), nitric oxide, and proinflammatory cytokines (such as TNF-*α* and IL-1*β*) is augmented in ZF rats [31, 32]. These findings are summarized in Table 1. Overall, in ZF rats, the cellular component of acquired immunity is impaired, while innate immunity is augmented.

A characteristic of OLETF rats is an age-dependent onset of diabetes [33]. OLETF rats also show augmented production of proinflammatory cytokines [34]. However, to the best of our knowledge, there have been no reports on acquired immune responses in OLETF rats.

Zucker strain rats were used for establishing Zucker lean (ZL) rats and Zucker diabetic fatty (ZDF) rats [35]. Insulin secretion is impaired in ZDF rats. Furthermore, ZDF rats were used for breeding with Wister rats and WBN/Kob rats, which were established to be Wistar fatty rats and WBN/Kob-Lepr (fa) rats, respectively [36–39]. These newly established rat strains are used in the investigation of diabetes complications.

### 4.2. Type 2 DM without Obesity

Several type 2 DM model rats exhibit glucose tolerance without obesity. These model rats exhibit mild hyposecretion (impaired secretion) of insulin from birth. Thus, energy metabolic efficiency is poor, and body weight is rather less than that of normal rats [40]. These models are termed nonobese type 2 DM model rats.

The Goto-Kakizaki (G-K) rat is a typical model rat of nonobese type 2 DM. G-K rats were established by selection of bred individuals exhibiting reduced glucose tolerance in Wistar rats [41, 42]. G-K rats already exhibit mild hyperglycemia and impaired insulin secretion in response to a glucose load at 4 weeks of age. In G-K rats, reduction and dysplasia of *β* cells in the islets of Langerhans are already observed at embryonic stages [43], and inflammation and fibrosis of the pancreas progress with age [44].

Presently, Rosengren et al. have demonstrated that overexpression of alpha2A adrenergic receptor (*α*(2A)AR) is the cause of hyposecretion of insulin in G-K rats [45]. Overexpression of *α*(2A)AR on the cell membrane has been shown to prevent membrane fusion during exocytosis in insulin secretion. In humans, a polymorphism of *α*(2A)AR gene (*ADRA2A)* was reportedly associated with an increased risk for type 2 DM [46].

G-K rats exhibit mild diabetic complications, such as retinopathy, nephropathy, and peripheral neuropathy [47]. Spontaneously Diabetic Torii (SDT) rats have severe diabetic complications without obesity [48–51]. Recently, mating of ZF rats with SDT rats has also been carried out: the mated rats exhibited an increased risk of hypertension caused by DM with obesity [52, 53]. To our knowledge, there have been no reports on immunological abnormality in SDT rats. The comparison between G-K rats and SDT rats is of interest for revealing the differences between them in terms of susceptibility to diabetic complications.

We investigated the immunological abnormalities of G-K rats, which are described in the following section.

### 4.3. Immunological Abnormalities of G-K Rats

We investigated immunological disorders in G-K rats, in comparison with Wistar rats [54–56]. The results are summarized in Table 1. There was no difference in the white blood cell counts between the G-K rats and Wistar rats. However, the T cell ratios in the white blood cells were increased, and B cell ratios were decreased in G-K rats, as compared with Wistar rats. The distinguishing feature of immunological abnormality in G-K rats is the impairment of the phagocytic activity of monocytes. Furthermore, natural (or innate) IgM production and Th2 immune reactivity were augmented in G-K rats, as compared with Wistar rats. These increases are diametrically opposed to the changes observed in DP-BB rats.

To clarify the abnormality of G-K rats, immunological parameters of peripheral blood from G-K rats were compared to the parameters of the blood from not only the Wister rat strain but also the Zucker rat strains, such as Zucker lean rats (ZL), Zucker fatty rats (ZF), and Zucker diabetic fatty rats (ZDF) (Table 2). Although the body weight of G-K rats gradually increased depending on their ages (~400 g), the insulin levels of G-K rats (1~3 ng/mL) at the nonfasting status were slightly higher than the levels of Wister rats. The insulin level of ZDF peaked at 8 weeks old (15~25 ng/mL) and then became lower after 16 weeks old (1~4 ng/mL), although the body weight of ZDF was saturated during 15 to 24 weeks old (350~450 g). After 16 weeks old, ZDF exhibited the most severe hyperglycemia (>600 mg/dL) among the Zucker rat strains. The blood glucose levels of G-K rats (200~300 mg/dL) were lower than those of ZF rats (300~400 mg/dL), and the body weights of ZDF rats were lower than those of ZL rats and Wister rats. Thus, both G-K rats and ZDF rats showed hyposecretion of insulin in response to blood glucose at 16 weeks old; however, there are great differences in the age-dependent changes of body weight, insulin level, and blood glucose level between G-K rats and ZDF rats.

The T cell ratios (% of CD3^+^) in ZDF rats were not significantly different from those of ZL or ZF rats. The T cell ratios of G-K rats were highest of the rat strains tested. Interestingly, the reaction to the pathogen (increased ERK activity upon lipopolysaccharide stimulation) of ZDF rats was the highest among the rat strains tested [57].

**Figure 1:**
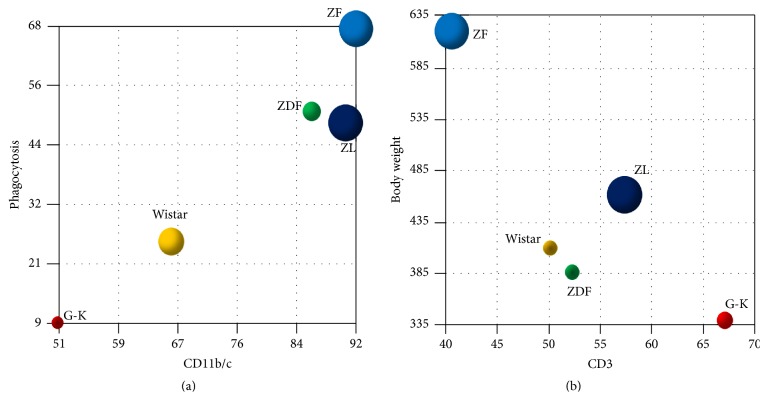
Relationships between phagocytosis and CD11b/c and between body weight and CD3 in the G-K/Wistar strains and Zucker strains. (a) Relationship between phagocytic ability and CD11b/c expression levels in G-K/Wistar and Zucker strain rats. (b) Relationship between body weight and CD3 ratio in lymphocytes in G-K/Wistar and Zucker strain rats. Bubble size indicates standard error, and bubble color shows each rat strain as follows: deep blue, ZL; light blue, ZF; green, ZDF; yellow, Wistar; red, G-K. The bubble charts were drawn using Graph-R software, version 2.19.

We analyzed the relationships between CD11b/c and phagocytic activity and between CD3 and body weight, in Zucker strains and G-K/Wistar strains of diabetic rat models (Figure 1). Phagocytic activity is associated with the expression level of CD11b/c (Figure 1(a)). The T cell ratio (% CD3^+^) in lymphocytes is inversely correlated with body weight (Figure 1(b)). Therefore, the measurements of CD3 on lymphocytes and CD11b/c on monocytes are useful for examining the relationship between immunity and diabetic episodes. For example, a case with CD3 upregulation and CD11b/c downregulation may be consistent with the pathologic status of G-K rats, and conversely, a case with CD3 downregulation and CD11b/c upregulation may be analogous to that of ZF rats. Further, a case with upregulation of CD11b/c and increased ERK activation corresponds to the pathologic status of ZDF rats.

## 5. Measurement of Monocyte Response and Amplification Circuits

Monocytes are important in the initiation of various immune responses [58, 59]. Monocytes migrate into tissues from the peripheral blood and are differentiated into inflammatory or regulatory macrophages depending on the environmental conditions [60–62]. Resident macrophages in various tissues migrate during the fetal period and proliferate in each tissue. However, up to half of the resident macrophages are derived from peripheral blood monocytes after birth [60]. Furthermore, infections or tissue injury augment monocyte migration into the tissue. Particularly, the intestine is known as “monocyte fall” [63]. The inflammatory response to enteric bacteria is regulated by monocyte migration into the intestine, but regulatory (anti-inflammatory) monocytes must also maintain intestinal homeostasis [63].

It is well known that proinflammatory responses are involved in the acceleration of arteriosclerosis and tolerance to insulin [64, 65]. In DM, high glucose blood is considered to indicate a mild inflammatory status [66, 67]. Indeed, the differentiation to M1 macrophages (inflammatory type macrophages) is augmented in adipose tissue or DM model mice [68, 69]. In contrast, chronic inflammation, such as tumor progression, induces immunosuppressive myeloid cells [70]. Thus, the physiological or pathological inflammatory status is important for predicting disease progression.

Monocyte status is regulated not only in the tissues, but also in the peripheral blood [71]. The interaction of monocytes with activated platelets induces proinflammatory responses [72], while the interaction of monocytes with “silent-platelets” accelerates anti-inflammatory responses [71]. Therefore, monocytes in the peripheral blood monitor systemic immunological conditions and regulate immune responses in various tissues. Thus, inflammatory monocytes or anti-inflammatory monocytes may become problematic.

The measurement of cytokine levels in the serum or cytokine mRNA in peripheral blood leukocytes is useful method for detection of immunological responses, but cytokine modulation in DM is much lower than in other immunological disorders. Thus, we measured signal transducers in peripheral blood monocytes. The assay to detect phosphorylated signal transducers in monocytes was conducted using heparinized blood and cell purification and long-term culture were not required. Additionally, the samples were stored at −20°C in methanol until the flow cytometry measurements were obtained. These advances simplify the procedure [55, 57, 73].

Immune systems are under the control of the NF-*κ*B and extracellular signal-regulated kinase families [65, 74]. Previously, we measured phosphorylated NF-*κ*B-p65 and extracellular signal-regulated kinase 1/2 (ERK) in peripheral monocytes from Zucker rats (Figure 3). Interestingly, the basal levels of phosphorylated NF-*κ*B-p65 in monocytes from ZF and ZDF were lower than that in monocytes from ZL (Figure 3(a)). Furthermore, the early induction of phosphorylated ERK by lipopolysaccharide at 10 min was significantly augmented in monocytes from ZDF compared to those from ZL and ZF (Figure 3(b)). According to the introduction of the Zucker strains from Charles River Laboratories, the insulin levels of ZL, ZF, and ZDF at 15~16 weeks old were 2.4 ± 0.7, 65.4 ± 37.1, and 6.3 ± 5.0 (ng/mL), respectively. On the other hand, the summated blood glucose levels in the glucose tolerance test (GTT) of ZL, ZF, and ZDF at 16 weeks old were 329 ± 15, 524 ± 64, and 2916 ± 594 (mg/dL), respectively. Thus, the insulin response is strongly attenuated in ZDF. These results suggest that deficiency of the leptin signal reduces basal NF-*κ*B activation and that insulin signal reduction augments ERK activation.

Collation of the signals to the amplification circuit indicated that the reduced basal level of NF-*κ*B was correlated with the reduction in the TCR repertoire and that augmentation of the ERK response was involved in the production of proinflammatory cytokines. These abnormalities of signal transduction in monocytes will be useful for clinical testing to evaluate the progression of DM related to immunological disorders.

## 6. Conclusion

Obesity is characterized by chronic and low-grade inflammation [75]. In recent years, free fatty acid-bound fetuin-A has been shown to be an endogenous ligand for toll-like receptor 4 (TLR4) [76], and hyperglycemia has been shown to enhance IL-1*β* secretion [77]. Furthermore, increasing knowledge of IL-1 family members leads to the clarification of the association between adipose tissue inflammation and insulin resistance [75]. IL-1*β* also directly contributes to dysfunction of *β* cells in islets [75]. These findings reveal the pathogenetic cascade, from obesity to DM, mediated by inflammation.

**Figure 2:**
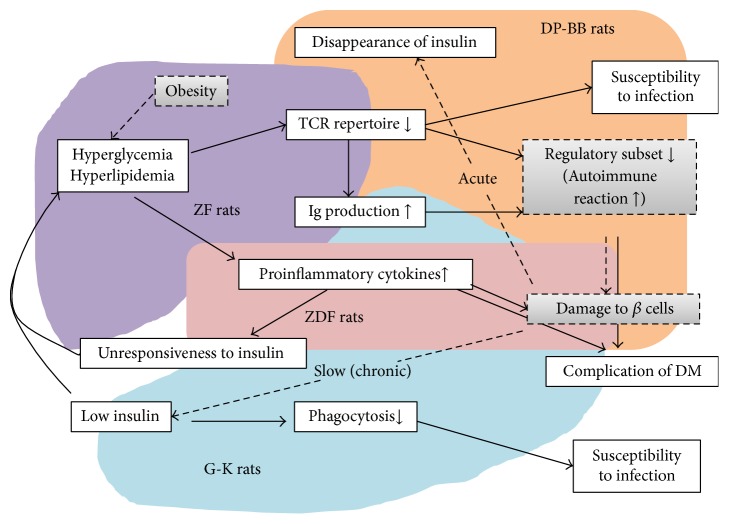
Pathogenesis of diabetes mellitus in rat models. Dash lines and gray squares indicate the pathogenesis of diabetes in each rat model. DM, diabetes mellitus; Ig, immunoglobulin; TCR, T cell receptor.

**Figure 3:**
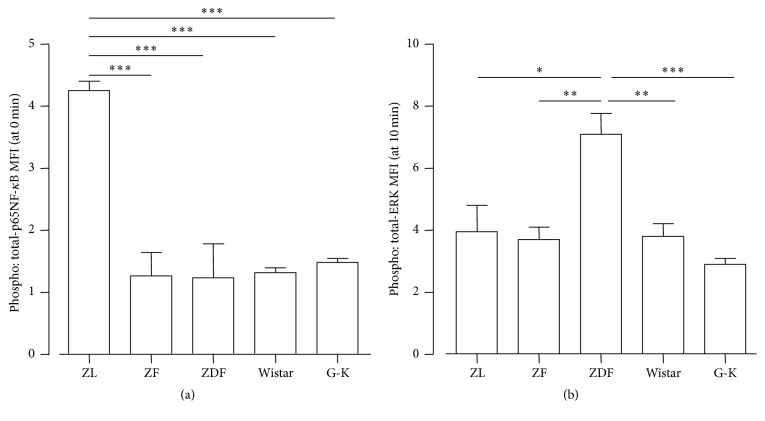
Difference in phosphorylation of signal transducers (NF-*κ*B and ERK) in monocytes dependent on diabetes status. The phosphorylation levels of NF-*κ*B and ERK in peripheral blood monocytes were measured by flow cytometry. Peripheral blood was collected from Zucker lean (ZL), Zucker fatty (ZF), Zucker diabetic fatty (ZDF), Wister, or G-K and stimulated with lipopolysaccharide (1 *μ*g/mL). After stimulation for 0 or 10 min, the blood was immediately fixed in Lyse/Fix Buffer (BD Biosciences) and treated with methanol at −20°C for membrane-permeabilization. Next, whole leukocytes in blood were stained with specific antibodies (anti-NF-*κ*B-p60 Abs and anti-ERK Abs from Cell Signaling Technologies). The cells were measured by flow cytometry, and the levels of total or phosphorylated signal transducers in monocytes were analyzed by monocyte-gating. The ratio of phosphorylation to the total was calculated based on the mean of fluorescence intensity. Changes in the phosphorylation levels of NF-*κ*B or ERK are shown in (a) or (b), respectively. Data are the mean ± SE (*n* = 3). ^*∗*^*p* < 0.05;  ^*∗∗*^*p* < 0.01;  ^*∗∗∗*^*p* < 0.001 (one-way ANOVA with post hoc test using Bonferroni).

Reduction and dysplasia of *β* cells in the islets are already observed at embryonic stages [43], based on data from G-K rats, suggesting that there is an amplification circuit in DM, as summarized in Figure 2. The background of each DM-rat model, shown as the circuit schema in Figure 2, is associated with immune responses. Autoimmune responses and lymphopenia are detected in DP-BB rats, lymphopenia and augmentation of humoral immunity are found in ZF rats, enhancement of the inflammatory response is detected in ZDF, and increased humoral immunity and reduction of phagocytic activity are observed in G-K rats. These rat models clearly illustrate that immunological phenomena are linked with the amplification circuit in DM.

In the future, the structure of DM-circuit consisting of diabetic episode and immune response will be clarified more in detail using DM-rat models.

## Figures and Tables

**Table 1:** Summary of immunological abnormalities in rat models of diabetes mellitus.

Cell type (subset)	Abnormality	Effect
*Alloxan-induced diabetic rats (type 1 diabetes mellitus)*		
T cells	Reduced T cell proliferation	Downregulation of cellular immunity and humoral immunity
Monocyte/macrophage	Impaired phagocytosis	(Susceptibility to infection?)

*DP-BB rat*		
CD4 helper T cells	T cell lymphopenia (Reduction of TCR repertoire)	Increase of autoreactivity
Th1	Increase of ratio	Augmentation of cellular immunity
Th17	Increase of ratio	Augmentation of inflammation
CD8 cytotoxic T cells	Decrease in cell number (Reduction in TCR repertoire)	Increase of autoreactivity

*Zucker fatty (ZF) rats*		
T cells	Decrease in cell number (CD4^+^ and CD8^+^)	Unknown (Impaired response?)
B cells	Augmentation of immunoglobulin (IgM, IgA, and IgG) production	Unknown (Autoreactive?)
Macrophages, (monocytes, dendritic cells)	Increase in cell number Augmentation of nitric oxide production Augmentation of inflammatory cytokine production	Augmentation of inflammatory responses

*G-K rats*		
T cells	Increase in cell ratio Th2 dominant	Unknown
B cells	Decrease in cell ratio Augmentation of natural IgM production	Unknown (Autoreactive?)
Monocytes	Attenuation of phagocytic activity	Unknown (Susceptibility to infection)

**Table 2:** Comparison of various parameters between the Zucker strains and G-K/Wistar strains.

Subject	ZL	ZF	ZDF	Wistar	G-K
Weight (g)	461.8 ± 31.2	620.8 ± 30.6^§^	387.6 ± 6.1	411.0 ± 7.9	340.2 ± 10.4^*∗*^
Glucose (mg/dL)	163.0 ± 27.8	399.0 ± 63.6^§^	636.6 ± 63.5^§^	111.2 ± 15.2	227.2 ± 31.8^*∗*^
CD3 (%)	57.5 ± 3.8	40.6 ± 5.2^§^	52.1 ± 1.8	50.1 ± 1.8	67.0 ± 1.4^*∗*^
CD45RA (%)	28.7 ± 3.1	33.9 ± 6.1	22.4 ± 1.7	32.7 ± 1.7	17.3 ± 1.5^*∗*^
CD11b/c (%)	90.9 ± 3.8	92.4 ± 1.6	86.4 ± 2.7	67.1 ± 4.3	51.6 ± 4.7^*∗*^
CD11b (%)	85.5 ± 3.8	70.8 ± 10.4	59.6 ± 10.3	67.8 ± 4.5	58.5 ± 4.3
Phagocytosis (MFI)	49.4 ± 20.6	68.1 ± 19.0	51.5 ± 8.8	25.7 ± 14.1	9.4 ± 0.5^*∗*^
pERK (ratio)	4.0 ± 0.9	3.7 ± 0.4	7.1 ± 0.7^§^	3.8 ± 0.4	2.9 ± 0.2

All data were obtained from 16-week-old, male rats.

ZL, Zucker lean rats; ZF, Zucker fatty rats; ZDF, Zucker diabetic fatty rats.

^§^ *p* < 0.05 (versus ZL rats, by one-way ANOVA, *n* = 4~5)

^*∗*^ *p* < 0.05 (versus Wistar rats, by Mann–Whitney test, *n* = 8~11)

CD3 (%) and CD45RA (%) indicate the corresponding ratios in lymphocytes.

Phagocytosis (MFI) indicates the phagocytic activity of monocytes using FITC-labeled BioParticle. MFI, mean of fluorescence intensity.

pERK (ratio) shows the ratio of phosphorylated ERK to total ERK in monocytes after stimulation with lipopolysaccharide for 10 min, as described in the text.
